# Six Types of Multistability in a Neuronal Model Based on Slow Calcium Current

**DOI:** 10.1371/journal.pone.0021782

**Published:** 2011-07-21

**Authors:** Tatiana Malashchenko, Andrey Shilnikov, Gennady Cymbalyuk

**Affiliations:** 1 Department of Physics and Astronomy, Georgia State University, Atlanta, Georgia, United States of America; 2 The Neuroscience Institute, Georgia State University, Atlanta, Georgia, United States of America; 3 Department of Mathematics and Statistics, Georgia State University, Atlanta, Georgia, United States of America; The University of Plymouth, United Kingdom

## Abstract

**Background:**

Multistability of oscillatory and silent regimes is a ubiquitous phenomenon exhibited by excitable systems such as neurons and cardiac cells. Multistability can play functional roles in short-term memory and maintaining posture. It seems to pose an evolutionary advantage for neurons which are part of multifunctional Central Pattern Generators to possess multistability. The mechanisms supporting multistability of bursting regimes are not well understood or classified.

**Methodology/Principal Findings:**

Our study is focused on determining the bio-physical mechanisms underlying different types of co-existence of the oscillatory and silent regimes observed in a neuronal model. We develop a low-dimensional model typifying the dynamics of a single leech heart interneuron. We carry out a bifurcation analysis of the model and show that it possesses six different types of multistability of dynamical regimes. These types are the co-existence of 1) bursting and silence, 2) tonic spiking and silence, 3) tonic spiking and subthreshold oscillations, 4) bursting and subthreshold oscillations, 5) bursting, subthreshold oscillations and silence, and 6) bursting and tonic spiking. These first five types of multistability occur due to the presence of a separating regime that is either a saddle periodic orbit or a saddle equilibrium. We found that the parameter range wherein multistability is observed is limited by the parameter values at which the separating regimes emerge and terminate.

**Conclusions:**

We developed a neuronal model which exhibits a rich variety of different types of multistability. We described a novel mechanism supporting the bistability of bursting and silence. This neuronal model provides a unique opportunity to study the dynamics of networks with neurons possessing different types of multistability.

## Introduction

Multistability is a fundamental attribute of the dynamics of neurons and neuronal networks [1]–[4]. As a feature it appears particularly advantageous for neurons which are part of multifunctional central pattern generators [5]–[7]. Some mechanisms underlying bistability, like the coexistence of tonic spiking and silence and the coexistence of tonic spiking and bursting, have been intensively studied and are well understood [8]–[12]. Surprisingly, there is a gap in our knowledge of the dynamical mechanisms supporting the bistability of bursting and hyperpolarized silence, bursting and subthreshold oscillations and multistability of bursting, subthreshold oscillations and silence. A classification of mechanisms supporting the multistability of oscillatory and silent regimes is yet incomplete, and remains a fundamental problem for both neuroscience and the theory of dynamical systems.

Bursting is a neuronal oscillatory activity consisting of groups of high-frequency spikes, “bursts”, separated by intervals of quiescence. It is a basic regime of neuronal activity, which for functionally different neurons can signify either a normal or a pathological state. It is a commonly recorded functional regime of activity of the neurons in the central pattern generators (CPGs): oscillatory neuronal networks executing the motor control of rhythmic movements, like breathing in mammals and the heartbeat in invertebrates such as the medicinal leech [13]–[16].

Bursting activity is the result of an interplay of ionic currents which are voltage-gated on various timescales. We envisage a neuron as a slow-fast dynamical system. The coexistence of different attracting regimes of activity, i.e., multistability, is not uncommon for such systems. The qualitative theory of dynamical systems provides a rigorous description of scenarios producing multistability of regimes in the system's dynamics. Exemplary studies by Rinzel (1978) and by Guttman, Lewis and Rinzel (1980) formulated and answered a set of questions which describe a basic scenario of bistability of tonic spiking and silence. It is based on the presence of a repelling periodic orbit separating the basin of attraction of the tonic spiking periodic orbit from the equilibrium representing the silent regime. The scenario also describes the modulation of the neuron's dynamics in response to variations of a bifurcation parameter. According to this scenario, the unstable limit cycle emerges through a subcritical Andronov-Hopf bifurcation and disappears through a saddle-node bifurcation for periodic orbits; both bifurcations define the boundaries for the bistability region. This gives rise to hysteresis and catastrophe-like, fast and non-reversible transitions between silence and tonic spiking as the bifurcation parameter is varied. Guttman, Lewis and Rinzel (1980) showed that a switch between these regimes can be executed by a pulse of current in experiments on the squid giant axon in saline with low calcium concentration.

This study is precipitated by our keen interest in the dynamics of the leech oscillator heart interneurons, which constitute the core of the leech heartbeat timing network. They are found as pairs of mutually inhibitory neurons located in ganglia 3 and 4 [16]. This preparation provides a unique opportunity for studying cellular and network mechanisms of bursting. A leech heart interneuron can be decoupled from its network with bicuculline [17]; its endogenous and network activity is well described by the canonical model [18], [19]. This model has been instrumental in predicting that these neurons have endogenous dynamics supporting bursting activity in a single cell, and has showed the high sensitivity of the bursting regime to variations of the leak current. This sensitivity explained why these interneurons show bursting activity while recorded extracellularly, and tonic spiking while recorded intracellularly. A rigorous analysis of the canonical model of a leech heart interneuron is a difficult subject. The model is a system of 14 ordinary differential equations with the variables operating on different time scales. We have numerically obtained a two-parameter bifurcation diagram, mapping oscillatory and stationary regimes on the plane of the leak current's parameters, conductance and the reversal potential of the leak current (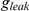,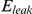). It shows the borders from silence to bursting and from bursting to silence, bounding the zone where bursting and silence coexist [19]. To demonstrate the bistability experimentally in a leech heart interneuron, and to identify the implications of this regime for operation of the leech heartbeat CPG, first we develop its low dimensional model and carry out the analysis of this model.

The reduced model introduced here is based on the fast sodium current, 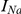; the slow, low-threshold calcium current, 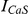; and the leak current, 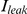. We have the following rationale for the choice of the currents. 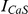 provides the slowest variable of the 14D canonical model of the leech heart interneuron. It has been suggested for the 14D model that 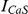 underlies the bursting activity [19]. Also, we have demonstrated that 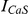 introduced through dynamic clamp can reinstate the bursting activity of a tonically spiking leech heart interneuron having all 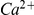-currents blocked and being isolated from other neurons by the application of saline containing 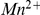 [20]. The significance of this achievement is apparent in light of past experiments showing that these neurons are sensitive to the leak current's parameters, so that we could not intracellularly record bursting activity of a neuron pharmacologically singled out from the network. We showed that an intrinsic mechanism for regulating burst duration might be based on the kinetics of 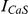 inactivation and we further corroborate that assertion in this study.

The model presented here makes a complementary study to our previous works on a simplified model since it employs the same fast subsystem supporting spiking as in Cymbalyuk and Calabrese, 2001, but differs in terms of its slow variable [12], [21]–[25]. That model represented a leech heart interneuron under a blockade of 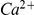 currents along with a partial block of outward currents. It included the fast sodium current, 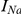, and non-inactivating slow potassium current, 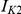, so that it was described by a system of three differential equations. The inactivation of 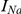 and membrane potential constituted the fast subsystem; and the activation of 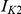 was the slow variable. The present model allows us to focus the investigation on the potential roles of 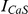 in the dynamics of a leech heart interneuron.

We construct a simplified model described by the set of ionic currents and their kinetics 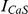, 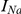. We sweep the parameters determining the kinetics of the currents and choose one set which produces activity with temporal characteristics close to those recorded experimentally from leech heart interneurons. Fitting the experimental data is not the primary goal of the article; we bring the model into the ballpark with the experimental observables through simple sweeps over parameters' values. We describe mechanisms supporting multistability in the model under variation of parameters of leak current.

## Materials and Methods

We present a simplified leech neuron model containing only the fast sodium (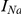) and slow calcium (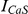) voltage-dependent currents and the leak current (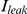). We refer to it as model {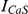 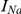}, according to the set of voltage-gated ionic currents which it contains. This model is described by the system of the following four equations:
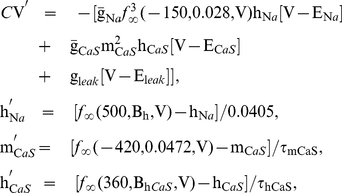
(1)where maximum conductances and reversal potentials of 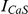 and 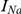 are 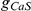 = 80 nS, 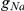 = 250 nS, 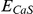 = 0.135 V, and 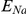 = 0.045 V, correspondingly; conductance, 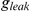, and reversal potential, 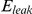, of the leak current are used as bifurcation parameters; C is the membrane capacitance, C = 0.5 nS. Function 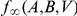 is a steady-state activation (inactivation) function of a voltage-gated ionic current given by
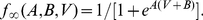
(2)Here 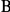 is the half-activation (half-inactivation) membrane potential at which 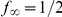. In the model, the activation of 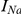 is considered to be instantaneous, so that 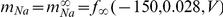. The voltage-dependent time constants for the activation and inactivation variables of the calcium current are taken from Hill et al. 2001:
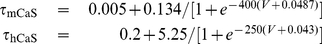
(3)One can see that the inactivation of the calcium current, 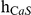, is the slowest variable in the model.

The bifurcation analysis was performed using the parameter continuation software CONTENT (Kuznetsov YA, Levitin VV, Skovoroda AR (1996) Continuation of stationary solutions to evolution problems in CONTENT. Report AMR9611. Amsterdam: Centrum/Voor Wiskunde en Informatica) freely available at http://www.staff.science.uu.nl/~kouzn101/CONTENT/. The solutions of this model were obtained using the Runge-Kutta method of the 4th order and a variable-order method based on numerical differentiation formulas, implemented as the ode15 s solver in Matlab (MathWorks, Inc.). Absolute and relative tolerances were set to 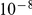 and 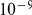, respectively.

Because the model is based on a subset of inward currents and hence lacks all outward currents except for the leak current, the balance of inward and outward currents has to be restored. It could be achieved in the sense that we tune up the available parameters so that the model produces the activity with the temporal characteristics of the bursting measured experimentally [19]; the instance of such a model found here we call the *canonical* model. We took into account basic temporal characteristics of the bursting waveforms such as the spike frequency, burst duration, interburst interval, period, number of spikes and duty cycle. By spike frequency we mean the average frequency of spikes in a burst. The burst duration is measured as the time between the first spike and the last spike in a burst. The interburst interval is the time between the last spike of a burst and the first spike of the following burst. The period of bursting is measured as the time between the first spike of a burst and the first spike of the consecutive burst, which is the sum of the burst duration and interburst interval. The duty cycle is the fraction of the period occupied by the burst duration, i.e. the ration of the burst duration to the period. To explore the waveforms of the bursting we swept values of half-inactivation voltages 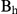 and 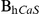 of the two voltage-gated currents. Then, the parameters determining the leak current, 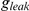 and 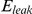, were used as the bifurcation parameters following Cymbalyuk et al., 2002.

### Canonical parameters

A considerable concern about the usability of this model was whether we could adjust it to produce bursting with characteristics close to experimentally recorded ones [19]. The targeted characteristics were: the burst duration was to be between 3.2 and 6.0 sec, the interburst interval was to be within 1.5 and 3.0 sec, the duty cycle was to be between 65.8 and 75.5%, the spike frequency was to be between 6.6 and 14.7 Hz. The model parameters supporting bursting with characteristics in the ballpark with these conditions were easily attained in three steps.

First, we varied the maximum conductances, and the inactivation kinetics for 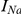 and 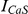. Relative to the 14D model, we set larger values for 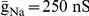, and 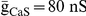 to make the corresponding currents more accentuated. Tuning the kinetic parameters 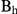 and 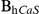 was motivated by the notion of the window mode of a voltage-gated ionic current [21], [26]. In this mode an inactivating current exhibits the properties of a persistent, non-inactivating current in the interval of membrane potentials where the steady-state activation and inactivation curves overlap. In the 4D model, the “window” mode of 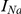 could play a role similar to that of the sodium persistent current, which supports burst duration in the canonical 14D model [18], [19]. A similar role would be played by the “window” mode of 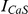.

From the studies of the 14D model we can infer that in the 4D model the activation of 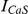 would be responsible for the inception of a burst, while the inactivation of 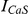 would control the burst termination. This mechanism of burst termination is similar to the one shown for the dynamics of a half-center oscillator assembled from two heart interneurons [18], [20].

**Figure 1:**
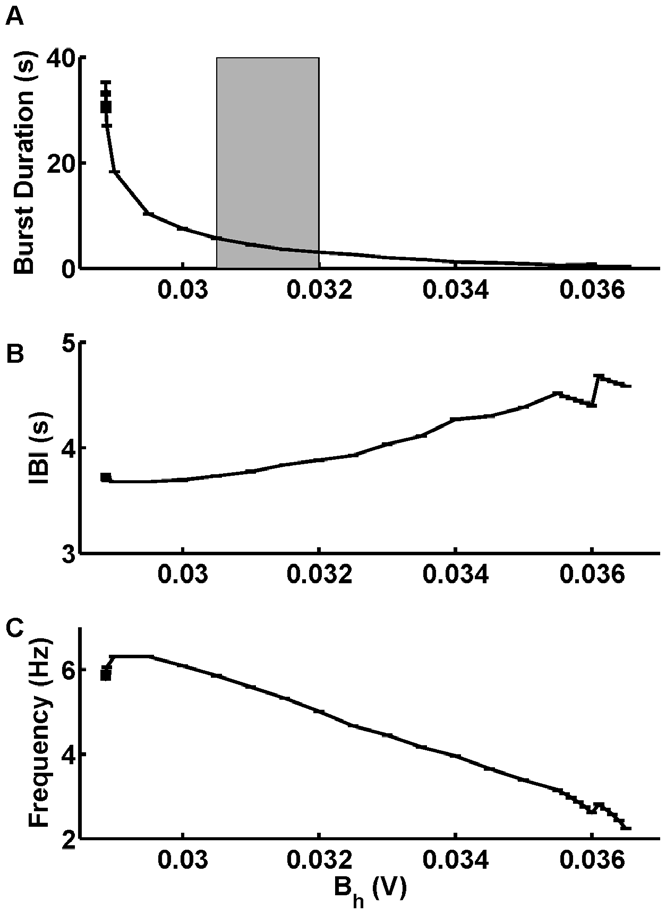
Dependence of temporal characteristics of bursting on half-inactivation voltage of 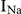. The variation of half-inactivation voltage of the fast sodium current, 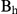, changes: (A) Burst duration, (B) interburst interval, and (C) spike frequency. This sweep of 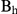 allowed us to adjust the burst duration so that it falls within the experimentally measured range, marked by the grey rectangle in A. The leak current parameters are 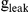 = 15.7 nS and 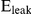 = −0.0505 V.

Second, we examined the activity of the model in response to variations of the half-inactivation voltage of 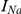, 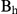, in the function 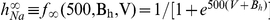. As 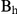 is increased, the curve 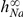 shifts towards the more hyperpolarized values, closing the window voltage interval. This manipulation of 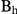 directly affects the steady-state conductance 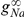, which produces the window current supporting the burst phase of the bursting activity. The overall decrease of 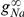 decreases the period, the burst duration and the duty cycle of the model (Fig. 1). The bursting activity occurs within the range of 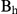 [0.02888 0.03692] V (Fig. 1). For some range of values of 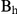 smaller than 0.02888 V, the model shows the tonic spiking activity. In the range of 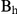 between 0.03692 V and 0.03790 V, the model exhibits the stable subthreshold oscillations; and it is silent for 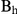 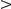
0.03790 V. The parameters, 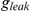 = 15.7 nS, 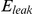 = −0.0505 V and 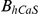 = 0.06 V fixed for this sweep, were chosen so that the model exhibits coexistence of bursting and silence. This sweep of 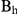 allowed us to adjust the burst duration so that it falls within the experimentally measured range, marked by the grey rectangle in Fig. 1 A, while the interburst interval and spike frequency were not attained. The discontinuity of the graphs of the interburst interval (Fig. 1 B) and the spike frequency (Fig. 1 C) near 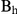 = 0.36 V is due to the integer-number difference in the number of spikes.

**Figure 2:**
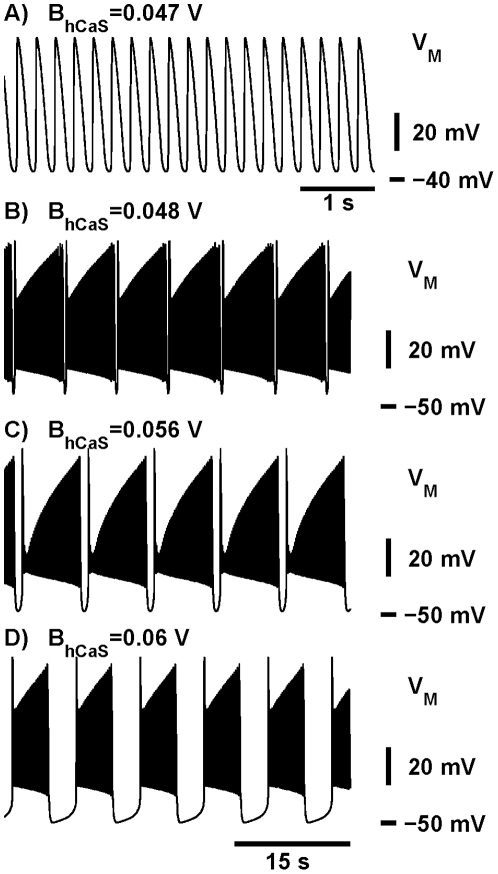
Transition from tonic spiking into bursting and the evolution of the bursting waveforms. The increase of the half-inactivation voltage of the slow calcium current, 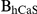 shifts 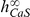 towards more hyperpolarized values of 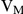 changing the activity from tonic spiking (A) to bursting (B–D). (A) For 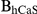 = 0.047 V the model exhibits a periodic tonic spiking activity. (B–D) The increase of 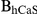 up to 0.048 V shifts 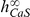 towards the hyperpolarized value of 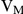 thus changing the activity from tonic spiking to bursting. (C) The increase of 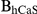 to 0.056 V expands the interburst interval. (D) 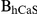 = 0.06 V brings the value of the interburst interval close to the targeted value. The leak current parameters are the same as in Fig. 1. 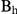 was 0.031 V for A and D. Panels (B)–(D) have the same time scale.

**Figure 3:**
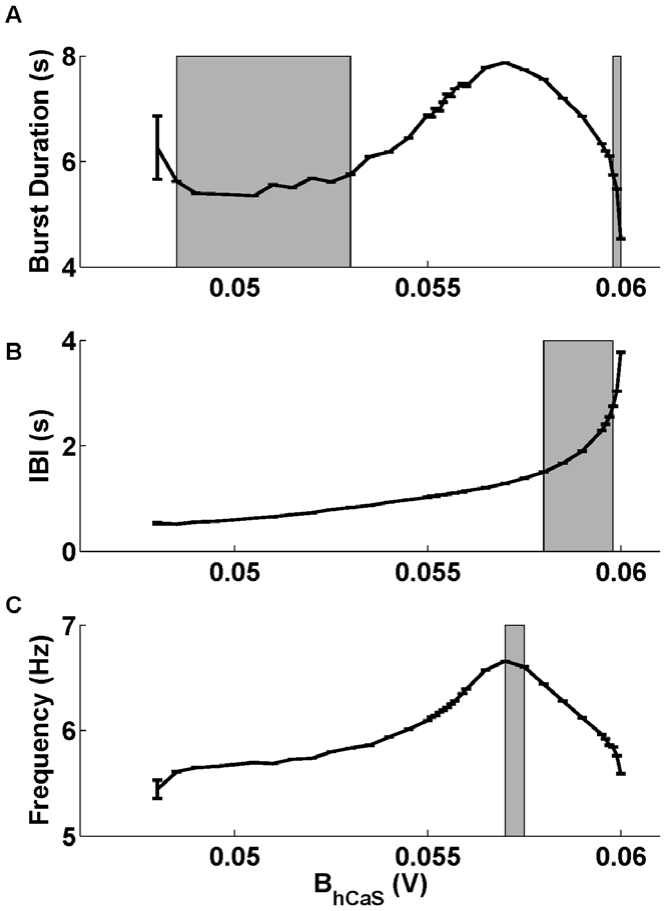
Dependence of temporal characteristics of bursting on the half-inactivation of 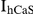. Dependencies of (A) the burst duration, (B) interburst interval and (C) spike frequency of the bursting activity on 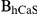 are shown. The shaded areas limit the values of 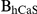 corresponding to the temporal characteristics of the activity measured experimentally. There are two ranges of values of 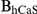, where the interburst interval falls within the scopes measured experimentally (the two gray rectangles in (A)). Other parameters are 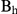 = 0.031 V, 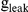 = 15.7 nS, 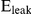 = −0.0505 V.

Third, to adjust the interburst interval, we swept the half-inactivation voltage of 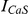, 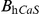. The increase of 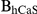 prolongs the interburst interval of the bursting activity. The interburst interval grows monotonically from 0.53 sec to 3.77 sec as 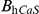 is swept from 0.048 V to 0.06 V (Figs. 2 and 3). The graph also shows the effect of variation of 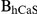 on the spike frequency within a burst (Fig. 3).

**Figure 4:**
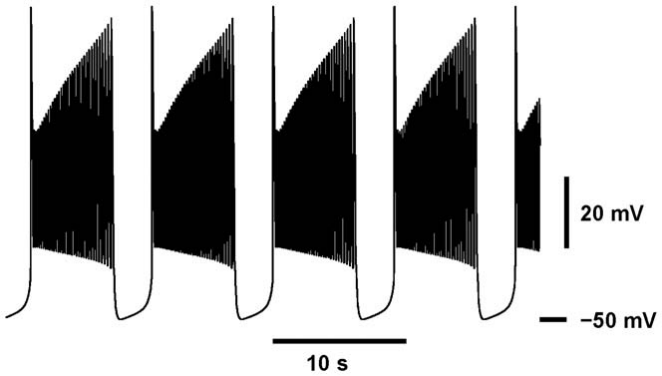
Bursting waveform with temporal characteristics close to experimental data. Bursting activity of the canonical 4D model at 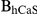 = 0.06 V, 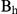 = 0.031 V, 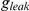 = 15.2 nS and 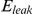 = −0.0505 V. The burst duration is 6.0 sec, teh interburst interval is 3.0 sec, the duty cycle is 66.4%, the number of spikes is 35, the frequency is 5.7 Hz.

Our parameter sweeps of the model were concluded with the following parameters 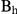 = 0.031 V, 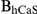 = 0.06 V. This adjusted model exhibits bursting activity with the burst duration of 4.5 sec, the interburst interval of 3.8 sec, the period 8.3 sec, and the duty cycle around 54.6%; the number of spikes per burst is 26, the spike frequency is 5.59 Hz (Fig. 2D). Leak current parameters are 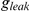 = 15.7 nS and 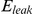 = −0.0505 V. We further tuned the model by setting 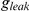 = 15.2 nS, so that the temporal characteristics of bursting activity fit well to the experimental data (Fig. 4).

## Results

### Equilibria and oscillatory regimes: the (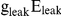)-parameter bifurcation diagram

**Figure 5:**
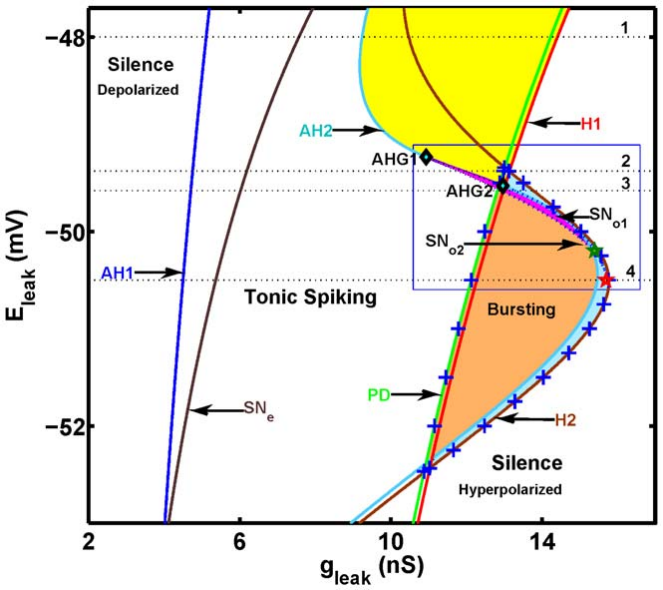
Two-parameter (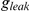,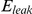) bifurcation diagram of the oscillatory and stationary regimes. The dark blue curve AH1 marks the supercritical Androvov-Hopf (A-H) bifurcation of the depolarized equilibrium. To the left of AH1, the equilibrium is stable. To the right, it becomes unstable, giving rise to stable tonic spiking. The orbit of spiking loses stability at the period doubling bifurcation, the green curve PD. Followed further, the tonic spiking periodic orbit disappears through a homoclinic bifurcation of an equilibrium marked by the red curve H1. The A-H bifurcation curve (AH2) for hyperpolarized equilibrium is shown in light blue. On this curve, the two points AHG1 and AHG2 mark the Bautin bifurcations (‘
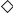
’). They bound the section of the curve where the A-H bifurcation is supercritical and gives rise to the stable subthreshold oscillations. The outer sections, above AHG1 and below AHG2, mark the subcritical A-H bifurcation, giving rise to the saddle orbit. The range where the saddle orbit exists is bounded by the homoclinic bifurcation of the saddle equilibrium, the light brown curve H2. Passage through the supercritical section leads to the onset of stable subthreshold oscillations. These oscillations vanish through a saddle-node bifurcation of periodic orbits on the dashed black curve 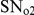 (Fig. 6). The area supporting bursting is obtained numerically (mapped in orange, light blue, and partially in pink) and its border is marked by ‘+’s. This border is bounded by the curves PD and H2. In the pink zone there coexist bursting and stable subthreshold oscillations (Fig. 8). The bright blue patch corresponds to the bistability of bursting and silence; it is bounded by the curves AH2, H2 and H1. The yellow area between the curves AH2 and PD corresponds to the coexistence of tonic spiking and the hyperpolarized silent regime. The dotted lines indicate the four levels of 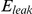 used in the diagrams: −0.048 V (1, Fig. 7-1), −0.04938 V (2, Fig. 7-2), −0.04958 V (3, Fig. 8-3), and −0.0505 V (4, Fig. 8-4). The dark brown curve, 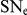, corresponds to the saddle-node bifurcation at which the hyperpolarized equilibria disappear (Figs. 7 and 8). The green ‘
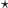
’ locates a point of tri-stability (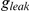 = 15.4 nS, 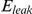 = −0.0502 V), illustrated in Fig. 10. The red ‘
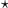
’ locates a point of bistability (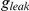 = 15.70 nS, 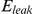 = −0.0505 V), shown in Fig. 11.

Parameters of the leak current are remarkable targets for modulation of neuronal excitability. For small values of the leak conductance the neuron stays silent at the depolarized equilibrium. This regime of depolarized silence describes a neuron in depolarization block. On the other hand, for large values of the conductance the neuron stays silent at the hyperpolarized equilibrium. The regime of hyperpolarized silence describes a neuron at a hyperpolarized rest state, or in hyperpolarization block. For different intermediate values of the conductance, it exhibits a plethora of oscillatory regimes such as various bursting, tonic spiking, and subthreshold oscillations. These regimes represent different levels of excitability. To inventory this rich variety of the neuronal dynamics, we created a two-parameter 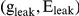 bifurcation diagram for equilibria and oscillatory regimes, which is shown in Fig. 5. On the diagram we map the areas of the hyperpolarized and depolarized silence (equilibria), the areas of tonic spiking (a stable periodic orbit), bursting activity, and various types of multistability. These areas are determined by these four types of codimension-one bifurcations: the Andronov-Hopf, the saddle-node and homoclinic bifurcations of equilibria, and the saddle-node bifurcations of periodic orbits.

**Figure 6:**
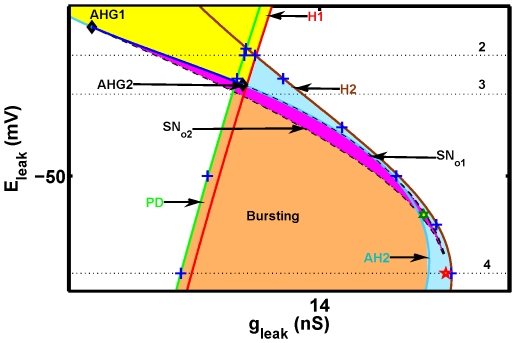
Inset from the (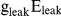) bifurcation diagram. The notations are the same as in Fig. 5.

**Figure 7:**
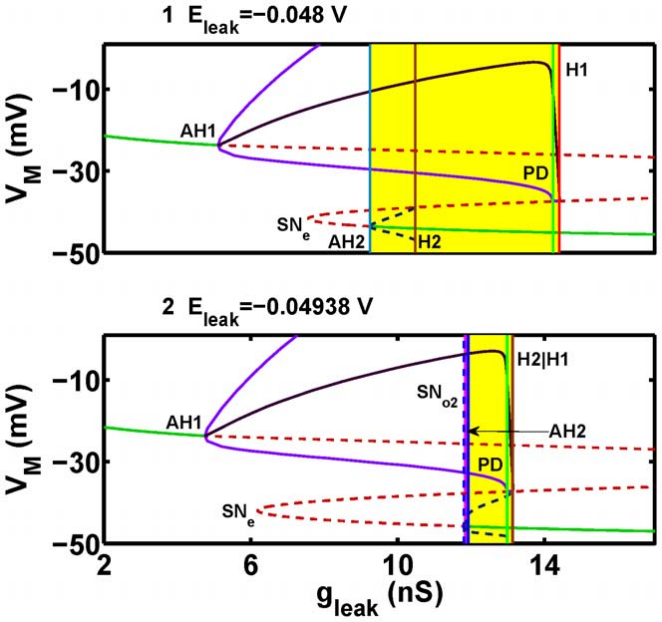
The one-parameter (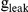) bifurcation diagrams corresponding to the levels 1 and 2 in Figure 5. (1) The diagram is constructed for 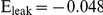 V and (2) for 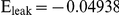 V. The solid green and dashed red curves represent the membrane potential at the equilibria, stable and unstable, correspondingly. Upper and lower branches correspond to the depolarized and hyperpolarized states of the interneuron. The branches are bridged by the middle saddle equilibrium, bounded by two folds corresponding to the saddle-node bifurcations. The tonic spiking emerges through a supercritical Andronov-Hopf bifurcation, AH1, that makes the depolarized branch unstable (dashed). Solid violet branches depict the minimum and maximum membrane potential values of the tonic spiking oscillations. The solid brown curve between them corresponds to the average membrane potential of the tonic spiking oscillations. The tonic spiking oscillations double their period after the period doubling bifurcation, PD (vertical green line), and disappear though the homoclinic bifurcation, H1. The hyperpolarized equilibrium loses stability though the Andronov-Hopf bifurcation (AH2): subcritical in (1) and supercritical in (2). (1): Unstable periodic orbit increases and terminates through a homoclinic bifurcation (H2). (2): The magnitude of stable oscillations increases as 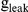 is decreased up to the saddle-node bifurcation, 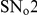. After the fold, the branch of unstable subthreshold oscillations is continued as 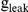 is increased until a homoclinic bifurcation (H2) where it terminates. The pair of dashed blue curves depicts the minimal and maximal values of the membrane potential of the unstable subthreshold oscillations. In both (1) and (2), the yellow rectangle indicates the range of bistability of tonic spiking and hyperpolarized quiescence. In (2) 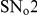 and AH2 bound the range of bistability of tonic spiking and the stable subthreshold oscillations (pink area).

**Figure 8:**
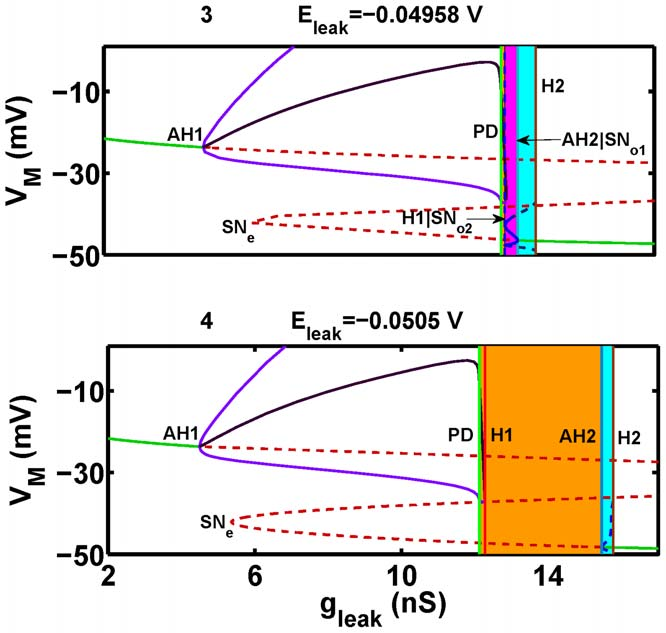
The one-parameter (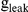) bifurcation diagrams corresponding to levels 3 and 4 in Figure 5. The diagrams show the evolution of equilibria and oscillatory regimes for two values of the leak reversal potential: 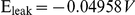 (3) and 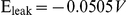 (4) plotted against the bifurcation parameter 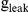. Labeling is the same as in Fig. 7. Numbers 3 and 4 correspond to the dashed lines 3 and 4 in Fig. 5. Here, the blue rectangles determine the range of bistability of the bursting and hyperpolarized equilibrium. In (3) the pink rectangle marks the range of the coexistence of bursting and the stable subthreshold oscillations. The critical values for the Andronov-Hopf bifurcation (AH2) and the saddle-node bifurcation 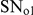 are very close to each other and marked by the single vertical line AH2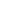 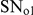.

The stable depolarized equilibrium loses stability through the supercritical Andronov-Hopf bifurcation. At the critical value, it gives rise to the stable periodic oscillations, see Figs. 5, 6, 7 and 8. This periodic orbit represents the tonic spiking activity of the neuron. The bifurcation occurs at the curve labeled AH1 in the 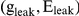 diagram in Fig. 5. At the bifurcation, the periodic orbit is born with zero magnitude and a non-zero frequency 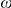, determined by the imaginary part of the characteristic exponents of the equilibrium state. The sign of first Lyapunov coefficient determines the stability of the new-born periodic orbit [27], [28]. It is negative for the supercritical Andronov-Hopf bifurcation. The corresponding bifurcation curve corresponds to the transition from depolarized silence into tonic spiking activity.

A large area in the middle of the bifurcation diagram corresponds to the tonic spiking regime of the model. As the parameter 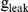 is increased from the bifurcation value, the magnitude of the stable periodic orbit increases. The orbit loses its stability through a period-doubling bifurcation on the curve PD in Fig. 5. With 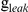 increased further, the unstable orbit disappears through the homoclinic bifurcation. At this value the orbit becomes a homoclinic loop of the saddle equilibrium. The bifurcation occurs with critical values of 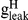 and 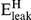 which are marked by the curve H1 on the diagram (Fig. 5). More precisely, to obtain this curve we exploited the fact that the period of the orbit grows as 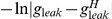 near the homoclinic bifurcation, thus it can be arbitrarily large in the vicinity of the bifurcation [27], [28]. The curve H1 marks the parameters' values corresponding to tonic spiking with a 25 second period. The area between the curves AH1 and PD supports the periodic tonic spiking in the model. Depending on the level, i.e., the value of the other parameter 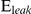, a further increase of 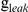 beyond the border H1 rightward leads to a transition from tonic spiking into either hyperpolarized silence or bursting (Fig. 5).

At a large value of 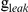 the neuron stays silent at the hyperpolarized equilibrium, which is a stable focus. Lowering 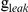 makes it unstable through another Andronov-Hopf bifurcation defining the curve AH2 in the parameter plane in Fig. 5. For the most part, the bifurcation is a subcritical one which means that it gives rise to an unstable, subthreshold periodic orbit of a saddle type. However, the segment on the curve AH2 bounded by the points labeled AHG1 and AHG2 corresponds to a supercritical Andronov-Hopf bifurcation, giving rise to the stable subthreshold oscillations. At these two points the first Lyapunov coefficient is zero. Each point locates a Bautin bifurcation, which locates the birth of the saddle-node periodic orbit with zero amplitude and non-zero frequency and has codimension 2. The feature of such a bifurcation is that its unfolding includes a bifurcation curve of saddle-node periodic orbits, that originates from the codimension 2 point. These two points lay on the intersection of the Andronov-Hopf bifurcation AH2 and the saddle-node bifurcation for periodic orbits 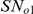 (Fig. 5 and Fig. 6). On the curve 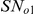, two subthreshold periodic orbits, stable and saddle, coalesce and vanish.

Outside the interval between AHG1 and AHG2, the periodic orbit emerges unstable through the subcritical Andronov-Hopf bifurcation at AH2. With 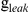 increased, it terminates at the homoclinic orbit of the saddle equilibrium. This event defines the curve H2 (Fig. 5, Fig. 6).

The bursting activity is observed in the model for quite a large range of the leak current parameter values. This range is bounded by ‘+’ in Fig. 5 and the boundaries of the area are special interest here. The onset of bursting appears to be in association with a rapid period doubling cascade leading to chaos in the model [29]. The final event of the scenario involves a homoclinic bifurcation of an unstable periodic orbit becoming the homoclinic loop of the saddle equilibrium. The reduced 4D model also demonstrates the coexistence of long-period, irregular bursting with chaotic tonic spiking oscillations. This bistability is observed in the narrow stripe bounded by the curves PD and H1. This type of transition has been described for the Hindmarsh-Rose model [30].

The model shows six types of multistability: (1) tonic spiking and the hyperpolarized silent regime; (2) tonic spiking and subthreshold oscillations; (3) tonic spiking and bursting; (4) bursting and subthreshold oscillations; (5) bursting and the hyperpolarized silent regime; (6) bursting, subthreshold oscillations, and silence.

**Figure 9:**
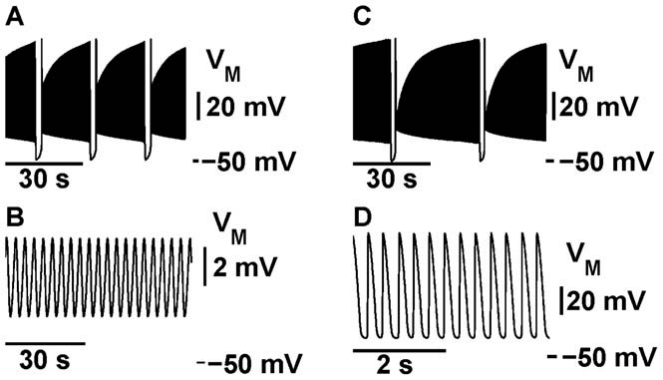
Examples of bistabilities of different oscillatory regimes. A–B) Voltage traces of coexistent bursting and subthreshold oscillations for 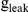 = 12.96 nS and 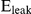 = −0.04958 V. C–D) Voltage traces of coexistent bursting and tonic spiking for 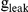 = 12.13195 nS and 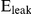 = −0.0505 V.

**Figure 10:**
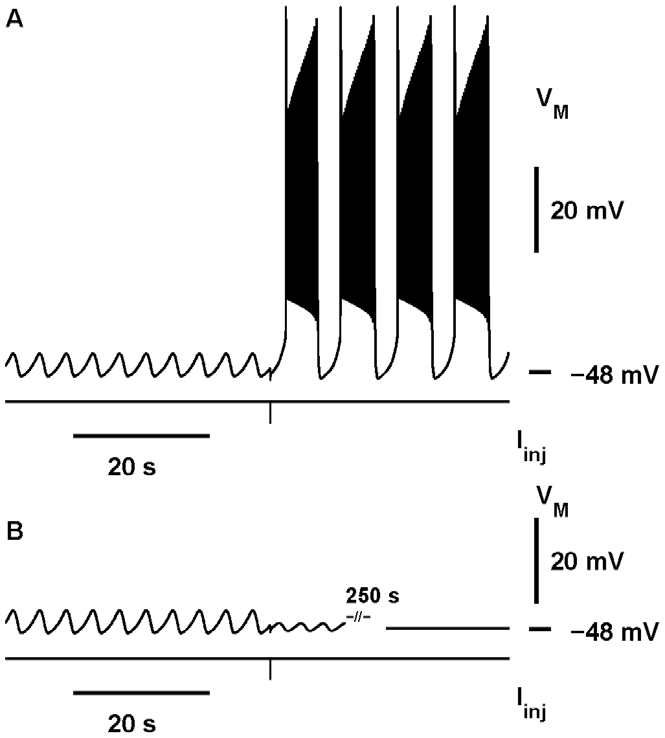
Multistability of three observable regimes. Stable subthreshold oscillations coexist with bursting and silent attractors, and by a pulse of current one can switch the activity from the subthreshold oscillations into either of the coexisting regimes. The parameters are 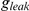 = 15.4 nS and 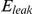 = −0.0502 V (marked by the green ‘
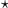
’ on Figs. 5 and 6). Initial conditions leading to the stable subthreshold oscillations presented are [V 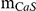 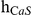 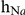] = [−0.04671933 0.5275212 0.01250879 0.9996319]. The pulses were delivered at 38.9 sec with the amplitudes −0.07 nA (A) and −0.047 nA (B).

**Figure 11:**
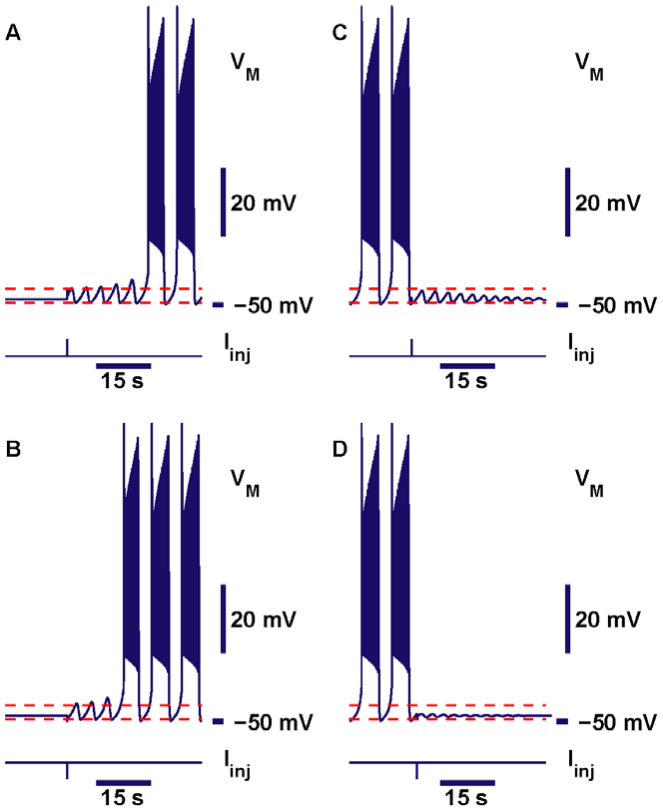
Bistability of bursting and silence. Examples of switches between bursting and silence produced by square pulses of injected current: switches from silence to bursting shown in panels (A,B) and switches from bursting to silence shown in panels (C,D). Stimulation of the neuron in the silent regime by a pulse of either depolarizing current, 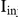 = 0.61 nA (A), or hyperpolarizing current, 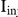 = −0.42 nA (B), switched the regimes. For this parameter regime, application of either a depolarizing (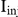 = 0.05 nA) (C) or hyperpolarizing pulse (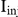 = −0.05 nA) (D) of the current could produce a switch from bursting into silence. The parameters of the model are 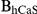 = 0.06 V, 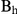 = 0.031 V, 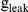 = 15.7 nS, 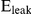 = −0.0505 V. The two red dashed lines mark maximal and minimal membrane potentials of the subthreshold oscillations.

To expand:

1. The basin of tonic spiking and the hyperpolarized silent state are separated by the stable manifold of the saddle equilibrium. In the diagram the zone of this bistability is bounded by the subcritical Andronov-Hopf bifurcation curve AH2, and the period-doubling bifurcation curve PD (Fig. 5, Fig. 7.1 for 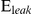 = −0.048 V).
2. The tonic spiking and subthreshold oscillations are separated by the stable manifold of the saddle equilibrium. This bistability area is determined by the range of parameter values supporting the stable subthreshold oscillations, i.e. it is limited by the saddle-node bifurcation curve for the periodic orbits,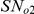 and the supercritical Andronov-Hopf bifurcation curve AH2 (Fig. 6, Fig. 7.2 for 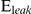 = −0.04938 V).
3. The tonic spiking and bursting coexist near the transition border between them (Fig. 5, Fig. 9 C–D). The mechanism underlying this type of multistability has been previously investigated in the Hindmarsh-Rose model [30].
4. The bursting and subthreshold oscillations are separated by the stable manifold of the unstable subthreshold oscillations. Similarly to (2), the area of this bistability is determined by the range of parameter values supporting the stable subthreshold oscillations, and hence is bounded by the saddle-node bifurcations' curves for the periodic orbits,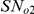 and 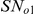 (Fig. 6, Fig. 8.3 for 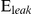 = −0.04958 V, Fig. 9 A–B).
5. The bursting and the hyperpolarized silent regime are separated by the stable manifold of the unstable subthreshold periodic orbit similar to (4). The corresponding zone is bounded by the range of parameters' values supporting the unstable subthreshold oscillations, i.e. the area is bounded by the subcritical Andronov-Hopf bifurcation curve AH2, and the homoclinic bifurcation curve H2 (Fig. 6, Fig. 8.4 for 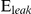 = −0.0505 V, Fig. 11).
6. The bursting, subthreshold oscillations and silence are separated by the stable manifolds of the two saddle orbits. One saddle orbit appears through the subcritical Andronov-Hopf bifurcation and disappears through a saddle-node bifurcation for orbits 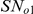. At 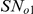 the stable subthreshold oscillations appear (the stable periodic orbit). In turn they disappear at a saddle node bifurcation 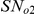, for a smaller value of the bifurcation parameter, where the second saddle orbit appears, which creates the barrier between the bursting and the stable subthreshold oscillations. The second unstable orbit disappears at the homoclinic bifurcation. Thus the tri-stability is bounded by either the Andronov-Hopf bifurcation or the second saddle-node bifurcation 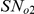 on one side and by the first saddle-node bifurcation on the other side 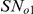. We illustrated the three regimes by switching neuron's activity from the subthreshold oscillations in either bursting or silent regimes (Fig. 10).

### Switching between bursting and silent regimes by a pulse of current

In the previous section, we applied the bifurcation analysis to find the range of parameters' values where bursting and silent regimes co-exist. The analysis predicts that a model with parameters taken from this range starting with different initial conditions would demonstrate one regime or the other. These results alone leave a concern as to whether these regimes would be observable. It might be difficult to demonstrate both regimes if the basin of attraction of one of them is too small. For example, for 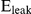 = −0.0505 V, the coexistence is determined for 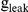 between 15.466 nS and 15.776 nS. If 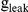 is picked in the vicinity of the Andronov-Hopf bifurcation, the basin of attraction to the equilibrium is small, and it is hard to achieve the switch from bursting into the silent regime. If 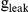 is picked close to the homoclinic bifurcation, then the situation reverses and it is harder to switch from the silent regime into bursting. With the following few numerical experiments we seek to test whether it is feasible to demonstrate both regimes. For this section, we have chosen a somewhat intermediate parameter value for the leak conductance 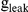 = 15.7 nS, with 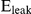 = −0.0505 V.

The question is, how to test for multistability? Although we address this question using a rather abstract model, we have a preference for those perturbations which can be easily implemented in an experimental set up, so that the predictions made could be tested in living neurons. A procedure of this sort has been utilized by Guttman, Lewis & Rinzel (1980). Following their approach, we used perturbations of the model made by “injection” of a square pulse of current into the neuron. We tested whether such perturbation could be used to switch from one regime to the other. The duration of the pulses was set to 0.03 sec, which is close to the effective width of a synaptic current pulse in the leech heart interneuron. We tried different amplitudes of the pulse. To switch from the silent regime into bursting, one needs to provide a pulse of current with a sufficiently large amplitude. The pulse can be either depolarizing or hyperpolarizing. For each polarity the critical value of the pulse which is just sufficient for the switch is different. Thus, there are two thresholds for the switch from silence into bursting. The description is more complicated for the switch from bursting into silence; and one more parameter of stimulation has to be taken into account. It is a phase within the period of bursting. Interestingly enough, for the parameters chosen one could switch from bursting into silence again by either a hyperpolarizing or depolarizing pulse of current (Fig. 11).

**Figure 12:**
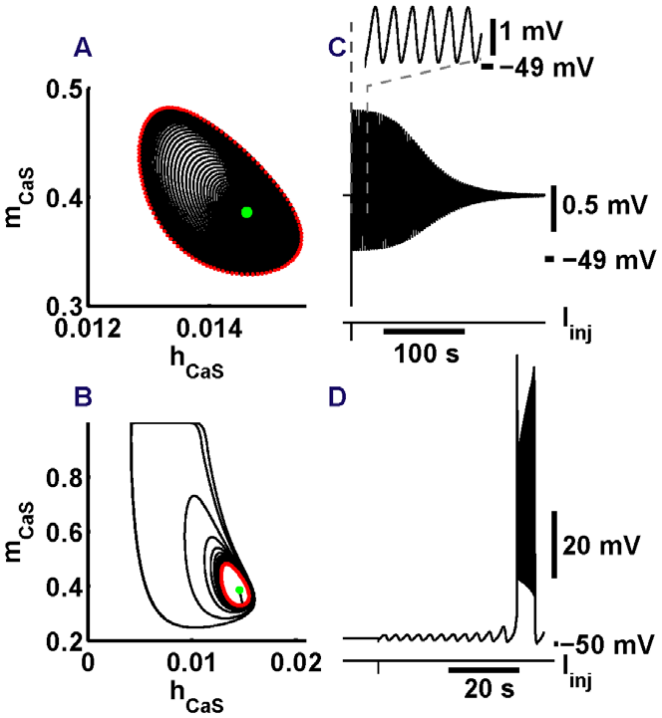
Numerical recording of unstable subthreshold oscillations. A pulse of current with a critical value of amplitude applied to a neuron in the silent regime allows recording oscillations in the close vicinity of the saddle periodic orbit. The parameters are the same as in Fig. 11 except for 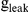 = 15.55 nS. The green dot represents the stable equilibrium point. The red dotted curve marks the unstable periodic orbit. Shown on the right are the corresponding traces after the pulses of magnitudes 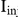 = −0.029 nA and 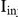 = −0.03 nA. The vertical dashed lines mark the time interval where the frequency of the unstable subthreshold oscillations and resonant frequency of the rest state are approximately the same, 1.97 rad/sec.

An important question for experimental testing is whether the unstable oscillations responsible for the bistability of bursting and silent regimes could be recorded. The model study predicts that it might be possible. A projection of a trajectory onto (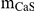, 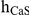)-plane before and after a pulse was injected into the neuron showed that the phase point oscillated in the vicinity of the unstable subthreshold orbit sufficiently long to trace a few cycles of the unstable oscillations (Fig. 12). Here, the green dot represents the stable hyperpolarized equilibrium, whose basin of attraction is bounded by the stable manifold of the unstable periodic orbit. The orbit is shown in red. Application of a hyperpolarizing 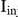 = −0.029 nA disturbs the neuron but the state is still in the basin of attraction of the equilibrium. However, application of a pulse of a larger magnitude 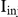 = −0.03 nA moves the model outside of the basin of the silent attractor away into bursting (Fig. 12). These two values specify a threshold in terms of the critical amplitude of the hyperpolarizing pulse of current.

## Discussion

Either a single neuron or neuronal networks can exhibit bistability as demonstrated in a number of theoretical and experimental studies. The coexistence of neuronal activity regimes - silence, subthreshold oscillations, tonic spiking and bursting - with each other has been observed in experimental studies [1], [9], [13], [31], [32]. Multistability has clear implications for dynamical memory and information processing in a neuron [3], [4], [31], [33]–[35]. In the area of motor control it could be a major mechanism of operation of multifunctional central pattern generators [5]–[7], [36]. Multistability can be classified according to regimes which coexist: coexistence of two silent regimes [1], coexistence of tonic spiking and silence [1], [2], [4], [8]–[37], coexistence of bursting and silence [19], coexistence of bursting and subthreshold oscillations [38], coexistence of different tonic spiking regimes [22], coexistence of different bursting regimes [19], [39], [40], and coexistence of bursting activity and tonic spiking [11], [12], [19], [32], [39]. One of the most studied types of bistability is the coexistence of tonic spiking and silence [8], [9]. In contrast, the coexistence of bursting and silence has not been the focus of any theoretical or experimental study of dynamics of a single neuron. Here we filled this gap in part by describing multiple scenarios of such coexistence in the dynamics of a low-dimensional model.

One of the first and most extensively studied types of bistability is the coexistence of tonic spiking and silence. The work by Rinzel (1978) set the standard for the investigation of bistability. It found the bistable properties of the Hodgkin-Huxley model of a squid giant axon. In this work, Rinzel formulated a set of questions which have to be answered in order to describe the mechanisms of bistability. What is the unstable, hyperbolic regime which separates the two observable, attracting regimes? How does this unstable regime appear and disappear as controlling bifurcation parameters are changed? Rinzel demonstrated that the unstable subthreshold oscillations are the regime which separates the tonic spiking periodic orbit from the equilibrium representing silence. The unstable oscillations are born through the Andronov-Hopf bifurcation and terminate at the saddle-node bifurcation for periodic orbits, where the stable orbit is the one corresponding to the tonic spiking. These two bifurcations define the range of the bistability in terms of the controlling parameter. Guttman et al. (1980) confirmed experimentally that the squid giant axon exhibits this type of bistability under low 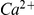 bath concentration. Repetitive firing and silence regimes can co-exist; and perturbation by a pulse of current can be used to switch between two regimes (Guttman et al., 1980). Bistability is of particular interest for motor control, since the coexistence of tonic spiking and silence has been shown in cerebellar Purkinje cells [4], [37] and has been induced by application of serotonin in spinal motoneurons from different species including the cat, rat, and mouse [1], [2].

In our previous studies, a 3D reduced model of the leech heart interneuron exhibited four types of bistability under different parameter regimes: (1) tonic spiking and bursting; (2) two different tonic spiking regimes; (3) tonic spiking and hyperpolarized silence; (4) bursting and depolarized silence [12], [22], [24]. In all four types, the regimes of activity are separated in the 3D phase space by the 2D stable manifold of the saddle type regime, either periodic orbit or equilibrium state. The topology of the manifold determines the threshold between the co-existing regimes.

Here, we developed a low dimensional, 4D model, which exhibits temporal characteristics close to those recorded from leech heart interneurons. Although the model is simple, it maintains a number of biophysical correspondences to the living counterpart. With this model we explored and accentuated the role of the low threshold slowly inactivating calcium current, as one sufficient to support a plethora of different mechanisms supporting the coexistence of different regimes of activity. It shows six types of multistability: (1) tonic spiking and the hyperpolarized silent regime; (2) tonic spiking and subthreshold oscillations; (3) tonic spiking and bursting; (4) bursting and subthreshold oscillations; (5) bursting and the hyperpolarized silent regime; and (6) bursting, subthreshold oscillations, and silence. We illustrated some of these mechanisms by a series of numerical experiments to show that they are sufficiently robust to be observed. We showed that switching between bursting activity and silence can be controlled by a pulse of current.

If we compare the lists of the mechanisms described for the two models, 3D and 4D, we observe one mechanism in common, the one underlying the co-existence of tonic spiking and hyperpolarized silent regimes which is based on a saddle equilibrium. We hypothesize that under different parameter regimes the two lists would record more mechanisms in common. The mechanisms of multistability described for these two models are generic and could be found in a variety of models under different parameter regimes.

Rhythmic motions of animals like swimming, crawling, walking, scratching, heart beating, and ingestion or rejection of food are controlled by specialized oscillatory neural networks, CPGs. It is a fundamental question whether each of these types of motion is controlled by a separate pattern generator. For a number of circuits it has been shown that some interneurons participate in multiple tasks, being part of a multifunctional central pattern generator [5]–[7], [41]. This solution appeals as much more parsimonious, and thus evolutionarily advantageous, compared to the one recruiting specialized circuits for each type of behavior. On the other hand, it is also appealing for mathematical modeling from the perspective of the theory of dynamical systems since the behavior of oscillatory neural networks, especially with heterogenous membrane and synaptic properties, is not well understood [24], [36]. Bistability as a membrane property of single neurons appears to be a natural feature of building blocks for such CPGs. In the future we plan to use this new model to explore the advantages and disadvantages of different types of multistability in small cental pattern generator circuits.

## References

[1] Hounsgaard J, Kiehn O (1989). Serotonin-induced bistability of turtle motoneurones caused by a nifedipine-sensitive calcium plateau potential.. J Physiol.

[2] Perrier JF, Hounsgaard J (2000). Development and regulation of response properties in spinal cord motoneurons.. Brain Res Bull.

[3] Heyward P, Ennis M, Keller A, Shipley MT (2001). Membrane Bistability in Olfactory Bulb Mitral Cells.. J Neurosci.

[4] Loewenstein Y, Mahon S, Chadderton P, Kitamura K, Sompolinsky H (2005). Bistability of cerebellar Purkinje cells modulated by sensory stimulation.. Nat Neurosci.

[5] Getting PA (1989). Emerging principles governing the operation of neural networks.. Ann Rev Neurosci.

[6] Marder E (1994). Invertebrate neurobiology. Polymorphic neural networks.. Curr Biol.

[7] Briggman KL, Kristan WB (2008). Multifunctional pattern-generating circuits.. Ann Rev Neurosci.

[8] Rinzel J (1978). On repetitive activity in nerve.. Fed Proc.

[9] Guttman R, Lewis S, Rinzel J (1980). Control of repetitive firing in squid axon membrane as a model for a neuroneoscillator.. J Physiol.

[10] Gutkin BS, Jost J, Tuckwell HC (2009). Inhibition of rhythmic neural spiking by noise: the occurrence of a minimum in activity with increasing noise.. Naturwissenschaften.

[11] Fröhlich F, Bazhenov M (2006). Coexistence of tonic firing and bursting in cortical neurons.. Phys Rev E.

[12] Shilnikov A, Calabrese R, Cymbalyuk G (2005). Mechanism of bi-stability: tonic spiking and bursting in a neuron model.. Phys Rev E.

[13] Marder E, Calabrese RL (1996). Principles of rhythmic motor pattern generation.. Physiol Rev.

[14] Ramirez JM, Tryba AK, Peña F (2004). Pacemaker neurons and neuronal networks: an integrative view.. Curr Opin Neurobiol.

[15] Smith JC, Abdala AP, Rybak IA, Paton JF (2009). Structural and functional architecture of respiratory networks in the mammalian brainstem.. Philos Trans R Soc Lond B Biol Sci.

[16] Calabrese RL, Nadim F, Olsen OH (1995). Heartbeat control in the medicinal leech: a model system for understanding the origin, coordination, and modulation of rhythmic motor patterns.. J Neurobiol.

[17] Schmidt J, Calabrese RL (1992). Evidence that acetylcholine is an inhibitory transmitter of heart interneurons in the leech.. J Exp Biol.

[18] Hill A, Lu J, Masino M, Olsen O, Calabrese RL (2001). A Model of a Segmental Oscillator in the Leech Heartbeat Neuronal Network.. J Comp Neurosci.

[19] Cymbalyuk GS, Gaudry Q, Masino MA, Calabrese RL (2002). Bursting in leech heart interneurons: cell autonomous and network based mechanisms.. J Neurosci.

[20] Olypher A, Cymbalyuk G, Calabrese RL (2006). Hybrid systems analysis of the control of burst duration by low-voltage-activated calcium current in leech heart interneurons.. J Neurophysiol.

[21] Cymbalyuk GS, Calabrese RL (2001). A model of slow plateau-like oscillations based upon the fast *Na*
^+^ current in a window mode.. Neurocomputing.

[22] Cymbaluyk G, Shilnikov AL (2005). Coexistence of tonic spiking oscillations in a leech neuron model.. J Comp Neurosci.

[23] Shilnikov A, Cymbalyuk G (2005). Transition between tonic spiking and bursting in a neuron model via the blue-sky catastrophe.. Phys Rev Lett.

[24] Shilnikov A, Gordon R, Belykh I (2008). Polyrhythmic synchronization in bursting networking motifs.. Chaos.

[25] Channell P, Cymbalyuk G, Shilnikov A (2007). Origin of bursting through homoclinic spike adding in a neuron model.. Phys Rev Lett.

[26] Hughes SW, Cope DW, Tóth TI, Williams SR, Crunelli V (1999). All thalamocortical neurones possess a T-type *Ca*
^2+^ “window” current that enables the expression of bistability-mediated.. J Physiol.

[27] Shilnikov LP, Shilnikov A, Turaev D, Chua L (1998). Methods of Qualitative Theory in Nonlinear Dynamics.. World Sci.

[28] Shilnikov LP, Shilnikov A, Turaev D, Chua L (2001). Methods of Qualitative Theory in Nonlinear Dynamics.. World Sci.

[29] Terman D (1992). The transition from bursting to continuous spiking in an excitable membrane model.. J Nonlinear Sci.

[30] Shilnikov AL, Kolomiets ML (2008). Methods of the qualitative theory for the Hindmarsh-Rose model: a case study- tutorial.. IJBC.

[31] Nadim F, Manor Y, Kopell N, Marder E (1999). Synaptic depression creates a switch that controls the frequency of an oscillatory circuit.. Proc Natl Acad Sci.

[32] Lechner H, Baxter D, Clark C, Byrne J (1996). Bistability and its regulation by serotonin in the endogenously bursting neuron R15 in Aplysia.. J Neurophysiol.

[33] Marder E, Abbott LF, Turrigiano GG, Liu Z, Golowasch J (1996). Memory from the dynamics of intrinsic membrane currents.. Proc Natl Acad Sci.

[34] Durstewitz D, Seamans JK (2006). Beyond bistability: biophysics and temporal dynamics of working memory.. Neurosci.

[35] Fall CP, Rinzel J (2006). An intracellular *Ca*
^2+^ subsystem as a biologically plausible source of intrinsic conditional bistability in a network model of working memory.. J Comp Neurosci.

[36] Venugopal S, Travers JB, Terman DH (2007). A computational model for motor pattern switching between taste-induced ingestion and rejection oromotor behaviors.. J Comput Neurosci.

[37] Williams SR, Christensen SR, Stuart GJ, Häusser M (2002). Membrane potential bistability is controlled by the hyperpolarization-activated current I(H) in rat cerebellar Purkinje neurons in vitro.. J Physiol.

[38] Wang XJ (1994). Multiple dynamical modes of thalamic relay neurons: rhythmic bursting and intermittent phase-locking.. Neurosci.

[39] Canavier CC, Baxter DA, Clark JW, Byrne JH (1994). Multiple modes of activity in a model neuron suggest a novel mechanism for the effects of neuromodulators.. J Neurophys.

[40] Butera RJ (1998). Multirhythmic bursting.. Chaos.

[41] Berkowitz A (2008). Physiology and morphology of shared and specialized spinal interneurons for locomotion and scratching.. J Neurophys.

